# Serum S100A12 and S100B proteins are independent predictors of the presence and severity of obstructive sleep apnea

**DOI:** 10.3906/sag-1806-147

**Published:** 2019-06-18

**Authors:** Gözde DEMİRCİ SAĞLAM, Adil ZAMANİ, Şebnem YOSUNKAYA, İbrahim KILINÇ

**Affiliations:** 1 Department of Chest Diseases, Sabuncuoğlu Şerafettin Educational Research Hospital, Amasya Turkey; 2 Department of Chest Diseases, Faculty of Meram Medical, Necmettin Erbakan University, Konya Turkey; 3 Department of Medical Biochemistry, Faculty of Meram Medical, Necmettin Erbakan University, Konya Turkey

**Keywords:** Obstructive sleep apnea, S100A12 protein, S100B protein

## Abstract

**Background/aim:**

Obstructive sleep apnea (OSA) is associated with serious cardiometabolic risks. Early diagnosis and treatment compliance are important. For this purpose, research is being carried out on biomarkers associated with the pathogenesis of the disease. We aimed to investigate whether serum S100A12 and S100B proteins could be used as biochemical markers in OSA patients to determine disease presence and severity.

**Materials and methods:**

A total of 60 (16 women, 44 men) patients with OSA and 50 (20 women, 30 men) controls were enrolled in this cross-sectional study. Each subject included in the study underwent full-night polysomnography (PSG). The presence and severity of OSA was assessed with the apnea–hypopnea index (AHI). In the OSA group, 17 cases were mild, 18 were moderate, and 25 were severe.****The serum levels of S100A12 and S100B were measured using the enzyme-linked immunosorbent assay (ELISA) technique. These protein levels were compared using Student’s t-test in the patient and control groups. Spearman’s rho correlation coefficients and corresponding P-values were calculated to determine the correlations between these protein levels and polysomnographic parameters. For evaluating the association between OSA and biomarkers, as well as possible confounding factors with S100A12 and S100B, we employed multiple linear regression analyses for the patients with OSA.

**Results:**

Serum levels of S100A12 and S100B were higher in patients than those in controls (P = 0.01* and P = *0.005, respectively), and a significant correlation was determined between S100A12 and S100B values and AHI (P = 0.0001; P = 0.0001), sleep time with SpO2 < 90% (P = 0.032; P = 0.01), minimum SpO2 during sleep (P = 0.019; P = 0.007), and oxygen desaturation index (ODI) (P = 0.001; P = 0.0001). In the linear regression analysis, AHI was independently related with both S100A12 (P < 0.0001) and S100B (P = 0.011). Receiving operating curves (ROC) identified patients with OSA: AUC for S100A12 = 0.643; AUC for S100B = 0.655 (P < 0.05).

**Conclusion:**

Serum levels of S100B and S100A proteins have high diagnostic performance in OSA and are independent predictors of OSA presence and severity.

## 1. Introduction

Obstructive sleep apnea (OSA) is characterized by intermittent hypoxemia and sleep disruption due to recurrent obstruction of the upper respiratory tract during sleep [1,2]. Due to this chronic intermittent hypoxemia and sleep disruption, OSA can trigger the development of systemic inflammation [3], oxidative stress [4], endothelial dysfunction [5], and metabolic syndrome [6]. Cardiovascular and cerebrovascular complications have been associated with oxidative stress and inflammatory reactions in OSA [7]. Moderate to severe sleep apnea was independently associated with a significantly increased risk of all-cause mortality in a study of a community-based sample [8]. Hence, early diagnosis and treatment compliance are important to prevent morbidity and mortality due to OSA. Polysomnography (PSG) enables the accurate diagnosis of OSA but is time-consuming. Therefore, OSA mostly remains undiagnosed [9]. Continuous positive airway pressure (CPAP) is highly efficacious, but a significant proportion of patients have poor adherence to therapy [10]. OSA-related biomarkers have the potential to provide information about the diagnosis, severity, prognosis, and treatment adherence of the disease [9]. To date, many inflammatory markers shown to be associated with OSA have been reported, such as C-reactive protein (CRP), tumor necrosis factor-alpha (TNFa), interleukin-6 (IL-6), IL-8, intercellular cell adhesion molecules (ICAMs), vascular cell adhesion molecules (VCAMs), and selectins [11]. However, their specificity and sensitivity have not been researched, nor have they been shown to be decisively reduced with treatment. In addition, the inflammatory markers have been shown to be influenced by many factors other than OSA. Therefore, there is still no ideal substance that can be used as a biomarker [9]. 

In clinical studies, it has been reported that the serum concentrations of calgranulins in the S100A12 and S100B proteins may be used as biological markers of cell stress [12]. There have been studies showing that in patients with OSA, an increase in these proteins may be associated with the disease [13–15]. S100A12 has been shown to be present on circulating leukocytes and is considered a susceptible marker for the local inflammatory process associated with oxidative stress [16]. S100A12 is a ligand for the receptor for advanced glycation end products (RAGE). RAGE expression has previously been shown to be affected by hypoxia via hypoxia inducible factor-1 [16]. RAGE plays a major role in inflammatory events, such as atherosclerosis [12]. Extracellular S100B interacts with RAGE and leads to nuclear factor-κB (NF-κB) activation. By binding and activating S100B, RAGE has been shown to induce inflammatory gene expression and facilitate oxidative inflammation [17]. In recent years, the S100B protein has been largely emphasized as the biochemical marker of cerebral diseases [18], and there are experimental animal studies demonstrate that S100B proteins induce similar intermittent hypoxia-associated neuronal degeneration as that seen in OSA [19]. 

This study aims to investigate whether serum S100A12 and S100B levels are associated with the presence and severity of OSA disease and whether these proteins may be biomarkers that reflect the presence and severity of OSA.

## 2. Materials and methods

This cross-sectional study was conducted between January 2015 and March 2016. 

### 2.1. Study group

Full-night PSG was performed for those who had been admitted to the sleep clinic (age range: 18-70 years). Patients were asked to provide a detailed medical history and underwent a basic medical examination for the following: weight, height, and heart and respiratory system inspection. The body mass index (BMI) was calculated by dividing the weight of the patient by the height squared (kg/m²). Comorbid disorders were identified by lung function tests, chest radiography, electrocardiography, and blood tests (liver and renal function tests, fasting blood sugar, blood fats, hemoglobin, hematocrit, sedimentation).

OSA was diagnosed when the patient’s apnea–hypopnea index (AHI) score was ≥ 15 events/h or ≥ 5 events/h with daytime sleepiness and/or the patient had habitual snoring or apnea complaints from a partner (n = 60). Subjects with AHI < 5 were included in the control group (n = 50). In total, 706 (566 OSA, 140 non-OSA) patients were examined with PSG in the sleep laboratory for 1 year, and 110 were included in the study. Patients with diagnoses of central sleep apnea or Cheyne-Stokes respiration (n = 7), the clinical manifestation of severe chronic obstructive pulmonary disease (COPD), or asthma (postbronchodilator FEV1 < 70% predicted) (n = 160) were excluded. Exclusions included a history of cerebrovascular or cardiovascular disease (n = 263), chronic renal insufficiency (19), diabetes (256), acute infection (5), rheumatologic disorder (15), or cirrhosis (3); 100 patients had both diabetes and vascular disease, 37 had both pulmonary disease and coronary artery disease, and 7 had all 3. Twelve patients did not agree to participate in the study. The protocol was approved by the Ethics Committee of Necmettin Erbakan University, Meram Medical School (number: 2015/183). Written informed consent was obtained from all subjects.

### 2.2. Polysomnography (PSG)

Full-night PSG was conducted for all subjects using a digital PSG system (Somnoscreen Plus, Somnomedics GmbH, Randersacker, Germany) in our sleep laboratory. PSG results were analyzed by a doctor according to the updated criteria of the American Academy of Sleep Medicine [20]. A reduction in peak signal excursions by ≥ 90% of preevent baseline for at least 10 s was considered apnea, a reduction in nasal pressure signal excursions by ≥ 30% of baseline together with arousal or ≥ 3% desaturation from preevent baseline for at least 10 s was considered hypopnea, and partial and/or complete airflow cessation with ongoing thoracoabdominal effort was considered an obstructive event. The number of apneas and/or hypopneas per hour of sleep during the study was defined as AHI. OSA severity was classified as mild (5 < AHI ≤ 15), moderate (15 < AHI < 30), or severe (AHI ≥ 30) [20]. 

During PSG, the following variables were recorded: sleep efficiency (SE), calculated as the total sleep time multiplied by time spent in bed; minimum oxygen saturation (minimum SpO2), the lowest oxygen saturation recorded during sleep; mean oxygen saturation (mean SpO2), the average oxygen saturation recorded during sleep; sleep time SpO2 < 90%, the amount of sleep time spent at < 90% of the oxygen saturation level; oxygen desaturation index (ODI), the number of ≥ 3% arterial oxygen desaturations per hour of sleep; rapid eye movement (REM) sleep ratio %, the ratio of REM sleep duration to total sleep duration; and NREM sleep ratio %, the ratio of nonREM3 sleep duration to total sleep duration.

### 2.3. S100A12 and S100B serum level assays

Blood samples from patients and controls were collected at the end of the PSG recording, between 07:00 and 08:00. Samples were centrifuged at 5000 g at 4 °C for 5 min within 1 h of collection. Serum was stored at –80 °C before further use, and all samples were processed in the same manner. S100A12 and S100B serum levels were measured by enzyme-linked immunosorbent assays (ELISAs), using commercially available kits for S100A12 and S100B proteins.

### 2.4. Statistical analysis

SPSS version 21.0 (SPSS Inc., Chicago, IL, USA) was used for the analysis. The Kolmogorov-Smirnov test was performed to determine if the continuous numerical variables were normally distributed. Group comparisons for variables that fit a normal distribution were performed by parametric methods. Student’s t-test for two independent group comparisons (i.e., the analysis of variance [ANOVA] for multiple group comparisons) was used. The Mann-Whitney U-test and the Kruskal-Wallis variance analysis were used for nonnormally distributed variables. Results are presented as median and interquartile range (IQR) values. In order to determine the correlations between categorical variables, the exact (Monte Carlo) corrected chi-square analysis method was preferred. Spearman’s rho correlation coefficients and corresponding P-values were calculated to determine the correlations between numerical variables. Ordinal logistic regression was executed to identify which of the predictors were significantly contributing to OSA severity. For evaluating the association between OSA and biomarkers, as well as possible confounding factors with S100A12 and S100B, we employed multiple linear regression analyses for the patients with OSA. In this analysis, we used serum levels of S100A12 and S100B as the dependent variables and evaluated the order of inclusion in the model of the following independent variables: age, sex, BMI, mean SpO2, ODI, sleep time with SpO2 < 90%, and AHI. A value of P < 0.05 was considered statistically significant. Sensitivity and specificity of S100A12 and S100B were based on the standard formulas of receiving operating curves (ROC). 

## 3. Results

The study group consisted of 110 participants including 60 OSA patients (16 women [26.7%] and 44 men [73.3%]) and 50 controls (20 women [40%] and 30 men [60%]). In the OSA group, 17 (28%) were mild, 18 (30%) were moderate, and 25 (42%) were severe.

There was no statistically significant difference between the groups regarding age or sex (P = 0.252, P = 0.152). The BMIs of patients with OSA were significantly higher than those of the control group (P = 0.002) (Table 1). There was no statistically significant difference between the groups in terms of SE (P = 0.256), but the other PSG data were statistically significantly different between the 2 groups. The values for minimum oxygen saturation, mean SpO2, sleep time with SpO2 < 90%, ODI, REM sleep duration, and NREM sleep duration were 82 (7.75) vs. 89 (3.50), 93 (4) vs. 94 (2), 5.50 (36.50) vs. 0 (2), 22 (95) vs. 5(1), 10 (15) vs. 8 (8.50), 3 (10) vs. 5 (11) in OSA and in the control groups, respectively (all P < 0.05).

**Table 1:** Clinical and biochemical characteristics of controls and OSA patients.

	OSA group Median (IQR)	Control group Median (IQR)	P
Age (year)	49 (18.50)	46 (23)	0.252
BMI (kg/m2)	30 (7)	28 (6.50)	0.002*
AHI (event/h)	24 (51.25)	5 (2.50)	0.000*
S.E. (%)	76 (19.75)	80 (14.50)	0.256
S100A12 ng/mL	421 (759.75)	314 (197)	0.001*
S100B pg/mL	358 (888)	241 (143)	0.005*

*P < 0.05. IQR: interquartile range, OSA: obstructive sleep apnea, BMI: body mass index, AHI: number of apneic hypopneas per hour, SE: sleep efficiency.

### 3.1. Serum S100A12 and S100B levels

**Figure 1:**
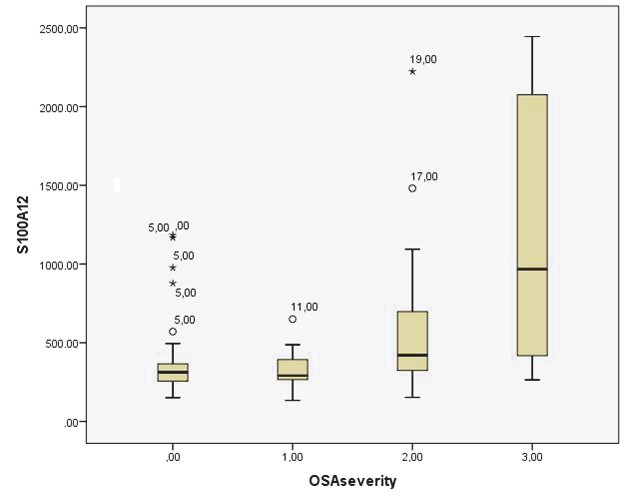
Serum S100A12 levels in control group and mild, moderate, and severe OSA patients.

**Figure 2:**
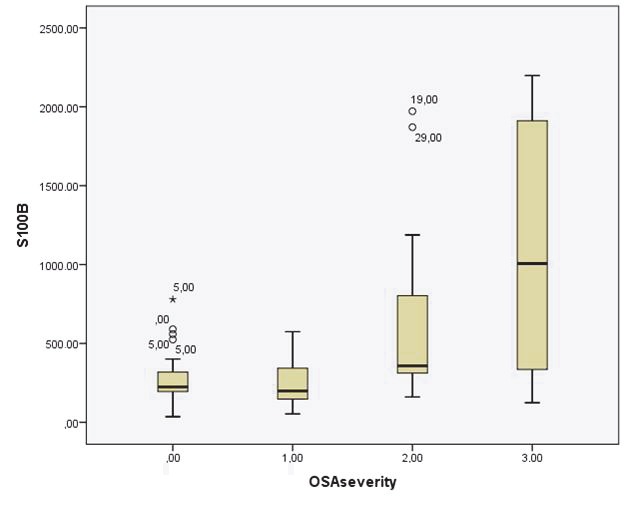
Serum S100B levels in control group and mild, moderate, and severe OSA patients.

S100A12 and S100B levels of patients with OSA were significantly higher than those in the control group (P = 0.001 and P = 0.005, respectively) (Table 1). Among the 3 subgroups of OSA patients, serum S100A12 levels rose with the severity of the disease (Figures 1–2). 

The control group’s S100A12 levels were mean 314 (IQR:201) ng/mL in males and mean IQR:321.50 (208.75) ng/mL in females. In the OSA group, S100A12 levels were mean 426 (IQR:1053.75) ng/mL in men and mean 314.50 (IQR:214.50) ng/mL in women. The control group’s S100B levels were mean 253 (IQR:159) pg/mL in males and mean 232.50 (IQR:147.75) pg/mL in females. The S100B levels in the OSA group were mean 384 (IQR:877) pg/mL in males and mean 317.50 (IQR:236) pg/mL in females. There was no statistically significant difference between the males and females in either group concerning S100A12 and S100B levels (P = 0.784 and P = 0.082, respectively and P = 0.903 and P = 0.12, respectively). 

### 3.2. Correlations

In the OSA group, a significant, moderate positive correlation was determined between the AHI scores and ODI with S100A12 (P = 0.0001; r = 0.525 and P = 0.001; r = 0.531) and S100B values (P = 0.0001; r = 0.531 and P = 0.0001; r = 0.520). In the study group, statistically significant weak positive correlation was observed between the time SpO2 < 90% values and S100A12 levels (P = 0.032; r = 0.277) and S100B levels (P = 0.01; r = 0.33). Additionally, in the study group, a statistically significant weak positive correlation was observed between the minimum SpO2 values and S100A12 levels (P = 0.019; r = –0.303) and S100B levels (P = 0.007; r = –0.345). There was no correlation between the REM and NREM3 sleep time ratios and the S100A12 or S100B protein serum levels (Table 2). Moreover, no significant correlation was detected between age and S100A12 (P = 0.760; r = –0.040) or S100B (P = 0.626; r = 0.064) proteins in the subjects included in the study. Ordinal logistic regression was executed to identify which of the predictors were significantly contributing to OSA severity. Age (P = 0.19, not significant), male sex (P = 0.007), BMI (P = 0.001), S100A12 (P = 0.0006), and S100B (P = 0.0002) were found to have significant effects on OSA severity. Odds ratios were 1.02 (95% confidence interval [CI]: 0.99–1.05), 3.55 (95% CI: 1.41–8.93), 1.14 (95% CI: 1.05–1.23), 1.001 (95% CI: 1.0–1.002), and 1.001 (95% CI: 1.001–1.002), respectively.

**Table 2:** The correlations of S100A12 and S100B levels with polysomnographic parameters in both groups.

		OSA group		Control group	
		S100A12	S100B	S100A12	S100B
AHI events/h	r	0.525	0.531	0.034	–0.098	p	0.0001*	0.0001*	0.818	0.503
	Minimum SpO2 (%)	r	–0.303	–0.345	0.042	0.080	p	0.019*	0.007*	0.775	0.584
Mean SpO2 (%)		r	–0.043	–0.125	0.047	0.002	p	0.745	0.340	0.751	0.991
	Time SpO2 <90% (%)	r	0.277	0.330	–0.203	–0.200	p	0.032*	0.010*	0.162	0.168
ODI (desaturation/h)		r	0.531	0.520	0.040	0.085	p	0.0001*	0.0001*	0.770	0.550
	REM sleep ratio (%)	r	–0.083	–0.187	0.187	0.115	p	0.530	0.152	0.199	0.433
NREM3 sleep ratio (%)		r	–0.082	–0.160	–0.207	–0.181	p	0.531	0.221	0.153	0.214
	BMI kg/m2	r	0.33	–0.52	0.223	0.208	p	803	697	0.87	0.110
S100A12 ng/mL		r	1	938	1	0.877	p		0.00*		0.00*

BMI: body mass index, AHI: number of apneic hypopneas per hour, ODI: oxygen desaturation index. *P < 0.05.

For evaluating the association between OSA and biomarkers, as well as possible confounding factors with S100A12 and S100B, we employed multiple linear regression analyses for the patients with OSA. According to this analysis, the main factor affecting S100A12 was found to be AHI (P = 0.002). Age, male sex, BMI, min. SpO2, ODI, and sleep time under 90% had no significant effect on S100A12 levels  Likewise, only AHI was a main factor affecting the level of S100B (P = 0.003) (Table 3).

**Table 3:** Multiple linear regression analyses predicting relation level of S100A12 and S100B with possible confounding factors in patients with OSA.

Parameter estimates for S100A12						Parameter estimates for S100B			
Parameter	DF	Estimate	Std. Er	t	Pr > |t|	Estimate	Std. Er	t	Pr > |t|
Intercept	1	1269.70	1487.4	–0.85	0.39	–359.79	1360.97	–0.26	0.79
Age(year)	1	2.78	5.05	0.55	0.58	1.67	4.62	0.36	0.71
Sex (male)	1	130.89	144.93	0.90	0.36	145.18	132.60	1.09	0.27
BMI (kg/m2)	1	8.39	12.86	0.65	0.51	–0.68	11.77	–0.06	0.95
Min SpO2 (%)	1	9.97	12.94	0.77	0.44	5.75	11.84	0.49	0.62
ODI (desaturation)/h	1	5.02	7.00	0.72	0.47	1.59	6.40	0.25	0.80
Sleep time <90%	1	1.07	3.42	0.31	0.75	2.25	3.13	0.72	0.47
AHI (event/h)	1	10.41	3.19	3.25	0.00*	9.03	2.92	3.09	0.00*

BMI: body mass index; AHI: number of apneic hypopneas per hour; ODI: oxygen desaturation index. Std. Er: Standard error. DF: degree of freedom. * P > |t| <0.05.

### 3.3. Positive and negative predictive values

**Figure 3:**
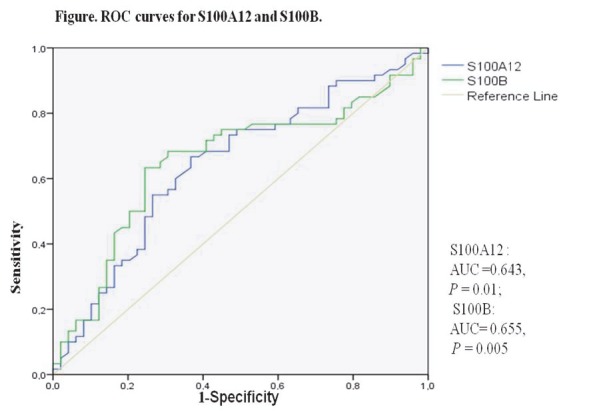
ROC curves for S100A12 and S100B.

In this study, when 315 ng/mL was used as a threshold for S100A12, the results obtained had a sensitivity of 75.0%, specificity of 51%, positive predictive value of 65.2%, negative predictive value of 62.5%, and accuracy of 64.2%. When the threshold used for S100B was 254 pg/mL, the results had 75.0% sensitivity, 55.1% specificity, 67.2% positive predictive value, 64.3% negative predictive value, and 66.05% accuracy. ROC curve identified patients with OSA significantly; the area under the curve (AUC) for S100A12 = 0.643, P = 0.01; AUC for S100B = 0.655, P = 0.005] (Figure 3).

### 3.4. Tables and figures

Table 1. Clinical and biochemical characteristics of controls and OSA patients. 

Table 2. Correlation of S100A12 and S100B levels with PSG parameters in both groups.

Table 3. Multiple linear regression analyses predicting relation level of S100A12 and S100B with possible confounding factors in the patients with OSA.

Figure 1. Serum S100A12 levels in control group and mild, moderate, severe OSA patients.

Figure 2. Serum S100B levels in control group and mild, moderate, severe OSA patients.

Figure 3. ROC curves for S100A12 and S100B.

## 4. Discussion

In this study, we detected that S100A12 and S100B levels were higher in the OSA group than in the control and that there was a positive correlation between OSA severity and these protein levels. According to our study, the minimum SpO2, sleep time SpO2 < 90%, and ODI values in the OSA group were correlated with the S100A12 and S100B values. These calcium-binding proteins from the S100 family have been shown to be present on circulating leukocytes and are considered to be susceptible markers for the inflammatory process associated with oxidative stress [16,21]. However, the main factor affecting the S100A12 level was AHI in our study. In addition, S100A12 levels correlated to the level of severity of disease in OSA patients. In a study conducted in China, it was found that S100A12 levels increased with increasing OSA severity. However, only men were included in the study, and the association between any PSG data other than AHI and the S100A12 protein level was not investigated [15]. In the OSA group, the origin of the S100A12 protein was reported to be upper airway inflammation.

Obesity is an important risk factor for OSA. OSA and obesity share common mechanisms such as: activation of inflammation, oxidative stress, and increased sympathetic activity [22]. In the study of Shi, the patient and control group BMIs were calculated as 26.48 ± 4.15 kg/m2 and 26.47 ± 2.38 kg/m2, respectively (P = 0.975); although there was no difference between groups in terms of BMI, S100A12 was found to be higher in the OSA group [15]. In our study, BMI averages in both OSA and control groups were overweight, but were significantly higher in the OSA group than in the control group. However, BMI was not found to be a significant predictor in the regression analysis established to estimate S100A12 or S100B levels.

The S100B Ca2+-binding protein has both intracellular and extracellular functions. RAGE has been recently shown to lead to NF-κB activation and facilitate oxidative events in monocytes, macrophages, microglia, and neutrophils by binding and activating S100B [17]. In our study, increasing levels of S100B showed a weak negative correlation with levels of oxygen saturation at night. However, in our study the main factor affecting the S100B level was AHI. Repeated sleep apneic episodes may lead to behavioral and morphological brain alterations in patients with OSA. A decrease in the gray matter volume in the right middle temporal gyrus in patients with OSA has been observed [23], and the same area was shown to be susceptible to hypoxic damage in an animal model of OSA [24]. An increase in S100B may reflect glial damage or reactive astrogliosis, an astrocytic reaction to neural injury, which may have neuroprotective properties [25]. S100B protein has been largely emphasized as the biochemical marker of cerebral diseases [18]. Our results are similar to the results of Riad et al. [26], who found that S100B serum levels were higher in patients than in controls, and that the level of S100B was correlated with AHI, lowest oxygen saturation, and ODI. In OSA, cerebral damage may occur following hypoxia. An animal study demonstrated that activation of the RAGE/NF-κB pathway induces neuronal degeneration, whereas activation of the S100B/RAGE/NF-κB pathway leads to reactive gliosis in the sleep apnea model of intermittent hypoxia exposure [19] Braga et al. [13] showed that there was an increase in S100B protein levels in OSA patients compared to a control group, but S100B did not correlate significantly with AHI score or minimum SpO2. In another study, Duru et al. [14] found that S100B levels were significantly higher in patients with OSA than in controls, but could not detect any significant correlations with AHI, minimum SpO2, mean SpO2, or sleep time SpO2 < 90% parameters.

Recently, Shih and Malhotra [27] described the properties required for an ideal biomarker for sleep apnea, suggesting that optimal biomarkers would be a tool for assessing diagnostic measures, burden, and severity of disease, as well as a method of measuring response to treatment [1]. If the biomarker is in a causal path known to be important in disease complications, changes in biomarker levels in response to treatment can be a reliable predictor of serious medical complications and allow the ideal biomarker to be used as a representative outcome measure in clinical trials. Two recent studies reported a significant decrease in serum S100B protein level with CPAP therapy in OSA patients [28,29]. 

In our study, when the thresholds used for S100A12 and S100B were 315 ng/mL and 254 pg/mL, respectively, the specificity and sensitivity were moderate, but when the AUC was examined, the ROC curve significantly identified patients with OSA. Similarly, in the study of Shi et al., S100A12 levels were reported to exhibit moderate sensitivity (83.33%) and specificity (66.22%) in showing the presence of OSA [15].

The confounding factors that are difficult to exclude in patients with OSA, such as age, BMI, and body fat mass, have been shown to play a role in many inflammatory markers previously shown to be associated with OSA.****A recent metaanalysis has shown that increases in inflammatory marker levels (including CRP, TNFa, IL-6, IL-8, ICAMs, VCAMs, and selectins) in OSA patients positively influenced the severity of the disease. However, a metaregression analysis has reported that age and BMI have moderate but significant effects on all inflammatory markers in OSA patients [11].

One limitation of our study was that the cognitive functions and daytime sleepiness of the patients were not investigated. An investigation of these conditions may give more information about the factors affecting S100B protein levels. Moreover, changes in the protein levels due to treatment were not evaluated. We included a selection of OSA population of both genders without serious comorbidities, but this population does not reflect the general population since patients with OSA have occasional comorbidities.

Our study suggests that serum levels of S100B and S100A12 proteins have diagnostic performance in OSA and are independent predictors of OSA presence and severity. In our study, it was found that age, gender, or BMI did not have an effect on S100A12 and S100B protein levels. This suggests that these proteins may be superior biomarkers to those previously described. These biomarkers also have the potential to improve assessments of prognoses and responses to treatment.

## Acknowledgments/disclaimers/conflict of interest

 This study was financially supported by the Necmettin Erbakan University Scientific Research Coordination Center (project number: 141518016).
